# Postresuscitation care with mild therapeutic hypothermia and coronary intervention after out-of-hospital cardiopulmonary resuscitation: a prospective registry analysis

**DOI:** 10.1186/cc10035

**Published:** 2011-02-14

**Authors:** Jan Thorsten Gräsner, Patrick Meybohm, Amke Caliebe, Bernd W Böttiger, Jan Wnent, Martin Messelken, Tanja Jantzen, Thorsten Zeng, Bernd Strickmann, Andreas Bohn, Hans Fischer, Jens Scholz, Matthias Fischer

**Affiliations:** 1 Department of Anaesthesiology and Intensive Care Medicine, University Hospital Schleswig-Holstein, Campus Kiel, Schwanenweg 21, Kiel 24105, Germany; 2 Institute of Medical Informatics and Statistics, Christian-Albrechts-University, Brunswiker Straße 10, Kiel 24105, Germany; 3 Department of Anaesthesiology and Intensive Care, University Hospital, Kerpener Straße 62, Cologne 50937, Germany; 4 Department of Anaesthesiology and Intensive Care, Klinikum am Eichert Eichertstraße 3, Postfach 660, Göppingen 73035, Germany; 5 Interhospital-Transfer-Service Mecklenburg-Vorpommern, German Red Cross, Moltkeplatz 3, Parchim 19370, Germany; 6 Department of Anaesthesiology and Intensive Care, Klinikum Lüneburg, Bögelstraße 1, Lüneburg 21339, Germany; 7 Department of Anaesthesiology, Klinikum Halle (Westfalen), Winnebrockstraße 1, Halle (Westfalen) 33790, Germany; 8 Department of Anaesthesiology and Intensive Care, University Hospital Muenster, Domagkstraße 5, Münster 48149, Germany; 9 Department of Anesthesiology and Intensive Care Medicine, University Hospital Tuebingen, Geissweg 3, Tuebingen 72076, Germany

## Abstract

**Introduction:**

Mild therapeutic hypothermia (MTH) has been shown to result in better neurological outcome after cardiopulmonary resuscitation. Percutaneous coronary intervention (PCI) may also be beneficial in patients after out-of-hospital cardiac arrest (OHCA).

**Methods:**

A selected cohort study of 2,973 prospectively documented adult OHCA patients within the German Resuscitation Registry between 2004 and 2010. Data were analyzed by backwards stepwise binary logistic regression to identify the impact of MTH and PCI on both 24-hour survival and neurological outcome that was based on cerebral performance category (CPC) at hospital discharge. Odds ratios (95% confidence intervals) were calculated adjusted for the following confounding factors: age, location of cardiac arrest, presumed etiology, bystander cardiopulmonary resuscitation, witnessing, first electrocardiogram rhythm, and thrombolysis.

**Results:**

The Preclinical care dataset included 2,973 OHCA patients with 44% initial return of spontaneous circulation (*n *= 1,302) and 35% hospital admissions (*n *= 1,040). Seven hundred and eleven out of these 1,040 OHCA patients (68%) were also registered within the Postresuscitation care dataset. Checking for completeness of datasets required the exclusion of 127 Postresuscitation care cases, leaving 584 patients with complete data for final analysis. In patients without PCI (*n *= 430), MTH was associated with increased 24-hour survival (8.24 (4.24 to 16.0), *P *< 0.001) and the proportion of patients with CPC 1 or CPC 2 at hospital discharge (2.13 (1.17 to 3.90), *P *< 0.05) as an independent factor. In normothermic patients (*n *= 405), PCI was independently associated with increased 24-hour survival (4.46 (2.26 to 8.81), *P *< 0.001) and CPC 1 or CPC 2 (10.81 (5.86 to 19.93), *P *< 0.001). Additional analysis of all patients (*n *= 584) revealed that 24-hour survival was increased by MTH (7.50 (4.12 to 13.65), *P *< 0.001) and PCI (3.88 (2.11 to 7.13), *P *< 0.001), while the proportion of patients with CPC 1 or CPC 2 was significantly increased by PCI (5.66 (3.54 to 9.03), *P *< 0.001) but not by MTH (1.27 (0.79 to 2.03), *P *= 0.33), although an unadjusted Fisher exact test suggested a significant effect of MTH (unadjusted odds ratio 1.83 (1.23 to 2.74), *P *< 0.05).

**Conclusions:**

PCI may be an independent predictor for good neurological outcome (CPC 1 or CPC 2) at hospital discharge. MTH was associated with better neurological outcome, although subsequent logistic regression analysis did not show statistical significance for MTH as an independent predictor for good neurological outcome. Thus, postresuscitation care on the basis of standardized protocols including coronary intervention and hypothermia may be beneficial after successful resuscitation. One of the main limitations may be a selection bias for patients subjected to PCI and MTH.

## Introduction

The initial success of cardiopulmonary resuscitation (CPR) in out-of-hospital cardiac arrest (OHCA) patients is influenced by numerous independent predictors - for example, patient-related factors, location of OHCA, presence of witnesses, willingness of bystanders to perform CPR attempts, and the initial electrocardiogram (ECG) rhythm [1-5].

Following initial successful CPR with return of spontaneous circulation (ROSC) but remaining comatose, mild therapeutic hypothermia (MTH) has been recommended [6,7] based on clinical studies reporting a better neurological outcome [8,9] and increased long-term survival rates [8].

In noncardiac arrest patients with acute myocardial ischemia, early reperfusion by either percutaneous coronary intervention (PCI) or pharmacological thrombolysis is recommended [10,11]. Since the underlying cause of OHCA is mostly cardiac arrhythmia and ongoing myocardial ischemia [12-14], therapeutic strategies of coronary reperfusion may be equally appropriate in OHCA patients. An international multicenter study of 1,050 OHCA patients, however, failed to demonstrate any benefit for systemic thrombolysis [15]. In contrast, Marcusohn and colleagues found improved short-term and longer-term survival with primary PCI after OHCA [16]. In addition, Wolfrum and colleagues have previously reported that MTH in combination with primary PCI is feasible and safe in patients resuscitated from OHCA following acute myocardial infarction [14]. More importantly, a standardized postresuscitation care bundle focusing on vital organ function - including MTH, liberal decision for PCI, and control of hemodynamics, blood glucose, ventilation and seizures - may be even more beneficial, as previously demonstrated [17]. Very recently, immediate PCI has been found to offer a survival benefit in a selected cohort of 435 patients with OHCA of presumed cardiac origin, regardless of the ECG pattern [18].

In an analysis based on the German Resuscitation Registry (GRR) [19], 2,973 patients were reviewed within the Preclinical care dataset; and 584 out of these 2,973 patients with additional documentation within a second database - the Postresuscitation care dataset -were analyzed with respect to the effects of MTH and primary PCI on 24-hour survival and neurological outcome at hospital discharge. We hypothesized that MTH and PCI would be independent prognostic factors for increased chance of 24-hour survival and good neurological outcome at hospital discharge. Regarding the Utstein recommendations, we analyzed both end points in our study; nevertheless, the relevant endpoint is most probably neurological outcome and survival status at hospital discharge.

## Materials and methods

The GRR is a Germany-wide prospective database for both OHCA and in-hospital cardiac arrest patients based on voluntary registration and documentation. The GRR is divided into two different datasets that can be analyzed separately.

The Preclinical care dataset records prehospital logistic issues, presumed etiology, resuscitation therapy and patient's initial outcome. Registration for the Preclinical care dataset was started in 1998.

The Postresuscitation care dataset is aimed at documentation of in-hospital postresuscitation efforts (for example, diagnostic procedures, hypothermia, and survival at hospital discharge) after hospital admission. The dataset includes exclusively OHCA patients from the Preclinical care dataset; however, registration for the Postresuscitation care dataset was started 6 years later, in 2004.

In the present study, the Preclinical care dataset included 2,973 prospectively documented OHCA patients with 44% initial ROSC (*n *= 1,302) and 35% hospital admission (*n *= 1,040) between 2004 and 1 July 2010. Seven hundred and eleven out of these 1,040 OHCA patients (68%) were also registered within the Postresuscitation care dataset. Data for the Preclinical care dataset have been allocated to the respective data of the Postresuscitation care dataset. Checking for completeness of both the Postresuscitation care and Preclinical care datasets required 127 Postresuscitation care cases to be excluded from further analysis, leaving 584 cases with complete data for final analysis.

Twenty-three emergency physician-staffed emergency medical systems were involved (GRR Study Group). The physicians were anesthetists, surgeons and cardiologists who had completed a special training program for emergency medicine.

The design and publication of the present study were approved by the scientific committee of the resuscitation registry of the German Society of Anaesthesiology and Intensive Care Medicine in compliance with current publication guidelines. Since cardiac arrest patients or their representative will mostly not be able to provide informed consent prior to treatment, the GRR is generally conducted under federal regulations that allow a waiver of informed consent comparable with the Resuscitation Outcomes Consortium funded by the National Heart, Lung, and Blood Institute of the National Institutes of Health. The Food and Drug Administration developed in 1996 specific regulations to permit research without prospective consent under carefully controlled circumstances. Secondly, any prerequisite condition of written informed consent for participation in the registry may lead to important additional selection biases.

### Inclusion criteria

The current study includes data from adult patients with OHCA, which was defined as the absence of signs of circulation and concomitant appearance of unconsciousness, apnea or gasping and pulselessness in accordance with the Utstein-style template [20]. After successful CPR, all patients were admitted to a hospital.

### Exclusion criteria

Patients with definite signs of death, patients with do-not-attempt-resuscitation orders, and patients presenting with injuries that were obviously associated with no chance of survival were excluded. In addition, patients initially resuscitated by basic life-support teams who subsequently did not receive any treatment from the advanced cardiac life-support team because the emergency physician decided to stop CPR due to pre-existing illness, medical history or after interviewing close relatives concerning the patient's supposedly negative intention for resuscitation were also excluded.

### Data management

We recently evaluated the establishment of the GRR to record both OHCA and in-hospital cardiac arrest [19]. The database has been proven congruent with the Utstein style, and control mechanisms have optimized data collection and data quality. The GRR is currently the largest resuscitation registry launched in Germany. The dataset was approved by the German Society of Cardiologists and Internal Medicine. The registry was also accepted and recommended both by the German Resuscitation Council and the German Society of Emergency Physicians, and in addition it represents the German database within the European Registry of Cardiac Arrest provided by the European Resuscitation Council.

The GRR is a prospective web-based database to register all emergency physician-related resuscitation efforts, as previously reported by our group [19].

### Definition of the datasets

The Preclinical care dataset originated from the Utstein-style templates, aiming at documentation of resuscitation efforts with 118 variables - in particular, prehospital logistic issues, presumed etiology, resuscitation therapy and patient's initial outcome.

The Postresuscitation care dataset also originated from the Utstein-style templates, aiming at documentation of postresuscitation efforts. The Postresuscitation care dataset includes demographic data, ECG, temperature management, cerebral performance category (CPC), hemodynamic variables, blood glucose level, circulatory support and diagnostic procedures (for example, chest X-ray scan, ultrasound, computer tomography, and survival at both 24 hours and at hospital discharge) [21]. In the present study, we focused on MTH (body temperature of 32 to 34°C) and on primary PCI performed within 24 hours after ROSC, although further details concerning MTH (for example, type of induction, type of cooling device, surface vs. intravascular, target temperature) and coronary intervention (for example, TIMI flow, type of stents, type of infarct, event-to-needle-time) were not registered within the GRR. Data from the Postresuscitation care dataset were reported to the resuscitation registry by the hospitals themselves. These data were also allocated to the respective Preclinical care dataset.

### Endpoints

In accordance with the Utstein definition, initial resuscitation success with ROSC was defined as a palpable pulse for more than 20 seconds.

The postresuscitation outcome was defined as 24-hour survival and neurological outcome at hospital discharge, since both endpoints represent variables within the Utstein style [20,22]. In the revised Utstein definitions from 2004 [20], 24-hour survival was downgraded from core to supplementary compared with the original 1991 version [22]. Nevertheless, both endpoints are still core variables within the GRR dataset.

Assessment of the neurological status was based on the CPC [23]. The performance categories are defined as follows: CPC 1, conscious and alert with normal function or only slight disability; CPC 2, conscious and alert with moderate disability; CPC 3, conscious with severe disability; CPC 4, comatose or in a persistent vegetative state; and CPC 5, certifiably brain dead or dead by traditional criteria. The best CPC score achieved at hospital discharge was used for calculation. A CPC score of 1 or 2 represents favorable functional neurological recovery because patients with these scores have sufficient cerebral function for independent activities of daily living, and was therefore defined as good neurological outcome. We state that most relevant endpoints are neurological outcome and survival status at hospital discharge. A CPC score of 3, 4, or 5 reflects unfavorable functional neurological recovery.

### Statistical analysis

With the exception of age, all data were binary or categorized variables. Outcome variables were analyzed employing Fisher's exact test, and the unadjusted odds ratio (OR) and 95% confidence interval were calculated. In addition, backwards stepwise (likelihood ratio) binary logistic regression analysis was used separately to identify the impact of MTH and PCI on the endpoints, respectively. We divided patients into the following subgroups: patients with/without any PCI, and patients with/without MTH. The following confounding factors were taken into account: age, location of OHCA, presumed etiology, bystander CPR, witnessing, first ECG rhythm and systemic thrombolysis. The adjusted OR and 95% confidence interval were calculated separately using binary regression analysis. The selected significance level was set at *P *≤ 0.05. SPSS version 17 (SPSS Inc., Chicago, IL, USA) was used for statistical analysis.

## Results

**Figure 1:**
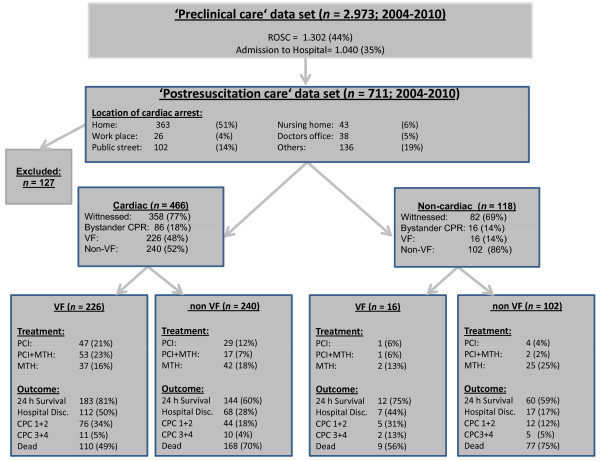
**Flow diagram of the study patients and outcomes**. CPC, cerebral performance category; CPR, cardiopulmonary resuscitation; MTH, mild therapeutic hypothermia; PCI, percutaneous coronary intervention; ROSC, indicates return of spontaneous circulation; VF, ventricular fibrillation.

Figure 1 shows a flow diagram of the study patients and outcomes. Of these patients, 396 were male and 188 were female. Mean (± standard deviation) age was 66 (± 18) years. The first monitored rhythm assessed by ECG revealed shockable rhythms (ventricular fibrillation (VF) or pulseless ventricular tachycardia (pVT)) in 242 patients (41%). OHCA was witnessed by bystanders in 324 patients (55%), and CPR was performed by bystanders in 102 patients (17%). The main cause of OHCA was presumably of cardiac origin in 466 patients (80%).

Table 1 shows the number of patients arranged by temperature management, by first ECG rhythm, and by coronary intervention with respect to hospital admission, 24-hour survival and good neurological outcome at hospital discharge.

**Table 1:** Subgroups of patients with hospital admission, 24-hour survival and good neurological outcome at hospital discharge

	Hospital admission (*n*)	24-hour survival		Good neurological outcome	
		*n*	%	*n*	%
Normothermia (total)	405	236	58	77	19
VF/pVT	150	108	72	39	26
Non-VF/pVT	255	128	50	38	15
PCI	81	67	83	44	54
No PCI	324	169	52	33	10
Hypothermia (total)	179	163	91	60	34
VF/pVT	95	87	92	42	44
Non-VF/pVT	84	76	90	18	21
PCI	73	70	96	36	49
No PCI	106	93	88	24	23

Subgroups of patients were arranged by: temperature management (normothermia vs. hypothermia), by first ECG rhythm (ventricular fibrillation (VF)/pulseless ventricular tachycardia (pVT) vs. non-VF/pVT), and by coronary intervention (percutaneous coronary intervention (PCI) performed within 24 hours after successful resuscitation vs. no PCI).

### Hypothermia in patients without coronary intervention

Out of 584 patients, 154 patients (26%) received PCI and 430 patients (74%) did not. In patients without PCI, MTH was associated with increased 24-hour survival (unadjusted OR 7.02 (3.7 to 13.3), *P *< 0.001) and good neurological outcome (unadjusted OR 2.21 (1.23 to 3.96), *P *< 0.01).

Binary logistic regression analysis confirmed that MTH (adjusted OR 8.24 (4.24 to 16.0), *P *< 0.001), bystander CPR (adjusted OR 3.25 (1.84 to 6.76), *P *< 0.001) and VF/pVT as first ECG rhythm (adjusted OR 1.96 (1.22 to 3.16), *P *< 0.01) were associated with improved 24-hour survival, whereas systemic thrombolysis was associated with worse chance of 24-hour survival (adjusted OR 0.52 (0.28 to 0.98), *P *< 0.05).

With respect to neurological outcome, regression analysis further revealed that MTH (adjusted OR 2.13 (1.17 to 3.90), *P *< 0.05), age <60 years (adjusted OR 2.25 (1.24 to 4.07), *P *< 0.01) and VF/pVT (adjusted OR 2.27 (1.26 to 4.09), *P *< 0.01) were independent factors for good neurological outcome at hospital discharge. Detailed results are presented in Table 2 and in Tables S1 and S2 in Additional file 1.

**Table 2:** Adjusted odds ratios for 24-hour survival and good neurological outcome from binary logistic regression analysis

	24-hour survival		Good neurological outcome	
	OR (95% CI)	*P *value	OR (95% CI)	*P *value
Patients without PCI (*n *= 430)				
Hypothermia	8.24 (4.24 to 16.0)	<0.001	2.13 (1.17 to 3.90)	0.014
Fibrinolysis	0.52 (0.28 to 0.98)	0.042	0.59 (0.25 to 1.38)	0.223
Location at home	1.42 (0.92 to 2.20)	0.116	1.59 (0.86 to 2.92)	0.137
Age <60 years	1.45 (0.89 to 2.36)	0.135	2.25 (1.24 to 4.07)	0.007
Witnessed OHCA	1.04 (0.64 to 1.68)	0.889	1.33 (0.66 to 2.69)	0.423
Bystander CPR	3.52 (1.84 to 6.76)	<0.001	1.45 (0.67 to 3.16)	0.35
VF/pVT as first ECG rhythm	1.96 (1.21 to 3.16)	0.006	2.27 (1.26 to 4.09)	0.006
Cardiac etiology	0.80 (0.47 to 1.34)	0.391	0.72 (0.36 to 1.50)	0.358
Patients with normothermia (*n *= 405)				
PCI	4.46 (2.26 to 8.80)	<0.001	10.81 (5.86 to 19.93)	<0.001
Fibrinolysis	0.32 (0.16 to 0.63)	<0.001	0.40 (0.15 to 1.05)	0.064
Location at home	1.39 (0.90 to 2.17)	0.141	1.44 (0.79 to 2.63)	0.237
Age <60 years	1.59 (0.98 to 2.58)	0.063	2.04 (1.10 to 3.78)	0.024
Witnessed OHCA	1.19 (0.73 to 1.93)	0.496	1.97 (0.92 to 4.21)	0.08
Bystander CPR	2.50 (1.34 to 4.69)	0.004	0.99 (0.44 to 2.24)	0.976
VF/pVT as first ECG rhythm	2.15 (1.33 to 3.48)	0.002	1.47 (0.79 to 2.73)	0.23
Cardiac etiology	1.06 (0.62 to 1.83)	0.821	1.37 (0.61 to 3.09)	0.445
All patients (*n *= 584)				
Hypothermia	7.50 (4.12 to 13.65)	<0.001	1.27 (0.79 to 2.03)	0.327
PCI	3.88 (2.11 to 7.13)	<0.001	5.66 (3.54 to 9.03)	<0.001
Fibrinolysis	0.39 (0.22 to 0.71)	<0.001	0.63 (9.33 to 1.22)	0.171
Location at home	1.29 (0.86 to 1.93)	0.223	1.03 (0.66 to 1.63)	0.888
Age <60 years	1.79 (1.14 to 2.82)	0.012	2.87 (1.83 to 4.49)	<0.001
Witnessed OHCA	1.31 (0.84 to 2.06)	0.237	1.83 (1.02 to 3.27)	0.042
Bystander CPR	2.27 (1.26 to 4.08)	0.006	0.72 (0.39 to 1.32)	0.287
VF/pVT as first ECG rhythm	1.81 (1.17 to 2.80)	0.008	1.61 (1.01 to 2.54)	0.043
Cardiac etiology	1.0 (0.61 to 1.64)	0.997	1.33 (0.70 to 2.50)	0.385

In contrast to Tables S1 to S6 in Additional file 1, which show detailed backward elimination starting with all candidate variables (step 1) and deleting any that were not significant (up to step 6), this table summarizes the adjusted odds ratio (OR) with 95% confidence interval (95% CI) from the respective step before deleting in the next step. Backwards stepwise binary logistic regression analysis was performed for 24-hour survival and good neurological outcome taking into account the following confounding factors: age, location of out-of-hospital cardiac arrest (OHCA), presumed etiology, bystander cardiopulmonary resuscitation (CPR), witnessing, first electrocardiogram (ECG) rhythm (ventricular fibrillation (VF)/pulseless ventricular tachycardia (pVT)), and systemic fibrinolysis. We performed three approaches for subgroup analysis by evaluating: hypothermia as an independent predictor in patients without any coronary intervention (*n *= 430), percutaneous coronary intervention (PCI) as an independent predictor in patients without hypothermia (*n *= 405), and the impact of both hypothermia and PCI in all patients (*n *= 584).

### Percutaneous coronary intervention in patients with normothermia

Out of 584 patients, 179 patients (31%) received MTH. In normothermic patients (*n *= 405; 69%), PCI was associated with increased 24-hour survival (unadjusted OR 5.06 (2.63 to 9.71), *P *< 0.001) and good neurological outcome (unadjusted OR 11.31 (6.25 to 20.47), *P *< 0.001).

Binary logistic regression analysis revealed that PCI (adjusted OR 4.46 (2.26 to 8.81), *P *< 0.001), bystander CPR (adjusted OR 2.50 (1.34 to 4.69), *P *< 0.01) and VF/pVT as first ECG rhythm (adjusted OR 2.15 (1.33 to 3.48), *P *< 0.01) were associated with improved 24-hour survival.

PCI (adjusted OR 10.81 (5.86 to 19.93), *P *< 0.001) and age <60 years (adjusted OR 2.04 (1.10 to 3.78, *P *< 0.05) were independent predictors of good neurological outcome. Detailed results are presented in Table 2 and in Tables S3 and S4 in Additional file 1.

### Combination of hypothermia and coronary intervention

To evaluate the combination of hypothermia and coronary intervention, we again performed unadjusted Fisher exact tests followed by adjusted regression analysis of the total group of 584 patients.

According to the Fisher exact test, MTH was associated with increased 24-hour survival (unadjusted OR 7.6 (4.32 to 13.37), *P *< 0.001) and good neurological outcome (unadjusted OR 1.83 (1.23 to 2.74), *P *< 0.01).

Following adjustment of these results by binary logistic regression, MTH (adjusted OR 7.50 (4.12 to 13.65), *P *< 0.001), PCI (adjusted OR 3.88 (2.11 to 7.13), *P *< 0.001), age <60 years (adjusted OR 1.79 (1.14 to 2.82), *P *< 0.05), bystander CPR (adjusted OR 2.27 (1.26 to 4.08), *P *< 0.01), and VF/pVT as first ECG rhythm (adjusted OR 1.81 (1.17 to 2.80), *P *< 0.01) were associated with improved 24-hour survival.

In terms of good neurological outcome at hospital discharge, PCI (adjusted OR 5.66 (3.54 to 9.03), *P *< 0.001), age <60 years (adjusted OR 2.87 (1.83 to 4.49), *P *< 0.001), witnessed OHCA (adjusted OR 1.83 (1.02 to 3.27), *P *< 0.05), and VF/pVT as first ECG rhythm (adjusted OR 1.61 (1.01 to 2.54), *P *< 0.05) were found to be independent predictors, whereas MTH (adjusted OR 1.27 (0.79 to 2.03), *P *= 0.33) did not improve outcome statistically significantly. Detailed results are presented in Table 2 and in Tables S5 and S6 in Additional file 1.

## Discussion

The present study focused on two therapeutic strategies - hypothermia and coronary intervention - after successful resuscitation from OHCA, and was based on the GRR database. In patients without any coronary intervention, MTH was associated with increased 24-hour survival and chance of good neurological outcome at hospital discharge. In normothermic patients, logistic regression analysis revealed that PCI was associated with increased 24-hour survival and the chance of good neurological outcome at hospital discharge. Owing to Utstein recommendations, the GRR dataset and the comparison with other scientific reports, we have analyzed both endpoints - 24-hour survival and neurological outcome at hospital discharge. Nevertheless, good neurological outcome at hospital discharge is reasonably the more relevant endpoint.

### Ventricular fibrillation/pulseless ventricular tachycardia as first ECG rhythm

In OHCA patients with VF/pVT as the first ECG rhythm, we found an increased 24-hour survival and a better neurological outcome at hospital discharge. An initial shockable ECG rhythm thus had a substantial influence on patient outcome. This result is in agreement with other studies [24-27]. The prevalence of VF/pVT as first rhythm has decreased in recent years, however, from 34% to 21% dependent on witnessing cardiac arrest and bystander CPR [28-32], but plausible explanation has not yet been found for this observation.

### Patient age

Although young adults are a minority among patients suffering from OHCA, these victims suffer from this catastrophic event when they are in a very active phase of life with a long life expectancy. Our registry analysis confirmed that patients aged <60 years had a better outcome in terms of good neurological outcome. We assume that most of the younger patients do not suffer from significant co-morbidities, and that the motivation of the medical team may be highest in these younger adults to make greatest efforts on any therapeutic option within the postresuscitation care period [33].

### Mild therapeutic hypothermia

MTH is currently a mainstay of postresuscitation care [6-9,34]. Most clinical investigations, however, mainly included patients with VF/pVT as the first ECG rhythm, reporting good neurological outcome in this subset of patients. In our database, MTH was associated with increased 24-hour survival. Interestingly, these favorable effects were observed irrespective of the initial ECG rhythm; the 24-hour survival rate was 92% in patients with VF/pVT and 90% in those with an initial nonshockable rhythm. More interestingly, 24-hour survival regarding hypothermia is rather questionable since the cooling therapy itself was still ongoing. Although 24-hour survival is mentioned as a core variable in the original Utstein style, it is still recommended as supplementary data in the Utstein update [20], and therefore should be reported as a clinical endpoint in these kinds of resuscitation registry analyses. Being aware of this limitation, we further analyzed survival and the proportion of patients with good neurological outcome at hospital discharge as the more relevant primary endpoint. MTH was associated with increased good neurological outcome at hospital discharge in patients without PCI.

### Coronary intervention

For the treatment of noncardiac arrest patients with myocardial ischemia, PCI is currently considered the treatment of first choice. But acute myocardial ischemia subsequent to coronary artery occlusion is also a common pathological correlate in cardiac arrest patients [35]. PCI has also been suggested to result in an increased chance of hospital survival in cardiac arrest patients suffering from myocardial ischemia. Gorjup and colleagues reported that OHCA patients with myocardial infarction may benefit from primary PCI similarly to noncardiac arrest patients with otherwise nonlethal myocardial infarction [36]. We are not, however, aware of any prospective randomized trial investigating the effect of primary PCI performed immediately after hospital admission in OHCA patients with successful CPR. Some smaller studies, however, have demonstrated beneficial effects of PCI in cardiac arrest patients [14,16,37].

In our registry analysis, PCI was an independent predictor of an increased chance of 24-hour survival and of good neurological outcome at hospital discharge. Our results revealed that the proportion of patients with CPC 1 or CPC 2 at hospital discharge increased from 10% to 54% in the group of normothermic patients if PCI was performed within 24 hours after ROSC. Interestingly, PCI was associated with increased 24-hour survival from 56% (159 out of 286 patients without PCI) to 88% (45 out of 51 patients with PCI) even in the subgroup of patients with an initial nonshockable rhythm. Patients with poorer baseline conditions (initial nonshockable rhythm) may thus also benefit from coronary intervention.

Our data may therefore support the hypothesis that a standardized postresuscitation care bundle, potentially including a liberal decision for coronary intervention, should be offered to most OHCA patients with successful resuscitation and hospital admission [17]. In addition, it should be noted that a typical history of coronary artery disease or ECG changes typical for ST-elevation myocardial infarction may be absent in up to 57% of OHCA patients, where coronary angiography revealed pathological findings with therapeutic options [35,38]. Further, clinical symptoms such as chest pain or risk factors often are lacking in the setting of OHCA. Comparable with severe trauma patients, therefore, prompt transfer after successful resuscitation to specialized hospitals/cardiac arrest centers may allow patients to benefit from this invasive therapeutic option [39]. This hypothesis is further supported by the findings of Dumas and colleagues, who recently demonstrated in a multivariable analysis of 435 prospectively registered patients that successful immediate coronary angioplasty was independently associated with improved hospital survival in patients with or without ST-segment elevation [18]. The high incidence of coronary lesions in the Parisian Region Out of Hospital Cardiac Arrest cohort study confirmed previous findings that link acute coronary syndrome and OHCA. Coronary plaque rupture or erosion, fragmentation, and embolization of thrombus were identified as factors able to trigger cardiac arrest. Similar rates have been noted in studies based on postmortem examination of patients with OHCA [40] or angiographic data [41].

### Postresuscitation care - combination of hypothermia and coronary intervention

Seventy-three patients received both MTH and PCI, irrespective of first ECG findings. Ninety-six per cent of these patients survived 24 hours and 49% were discharged with CPC 1 or CPC 2 compared with 54% and 11% of patients without any therapeutic procedure, respectively. The proportion of patients with good neurological outcome at hospital discharge was thus much higher in patients receiving both forms of treatment compared with normothermic patients without PCI. We therefore suggest that a therapeutic bundle of hypothermia and coronary intervention in addition to standard critical care may be beneficial in selected successfully resuscitated patients. We are not aware of any randomized controlled study investigating the therapeutic approach of a combination of hypothermia and coronary intervention. A few small clinical studies including historical control groups and case reports, however, have recently indicated that the combination may be feasible and may indeed be associated with benefits for the individual patient [14,17,42,43].

Considering the combination of MTH and PCI, we performed binary logistic regression analysis including all patients (*n *= 584). Both MTH and PCI were independently associated with increased 24-hour survival (MTH adjusted OR 7.50 (4.12 to 13.65), and PCI adjusted OR 3.88 (2.11 to 7.13)). In terms of neurological outcome at hospital discharge, however, only PCI was independently associated with increased chance of good outcome (adjusted OR 5.66 (3.54 to 9.03)). Although MTH was significantly associated with good neurological outcome in 44% and 21% of patients with VF/pVT and non-VF/pVT in contrast to 26% and 15% of normothermic patients, respectively (unadjusted OR 1.83 (1.23 to 2.74), *P *< 0.05), statistical significance was not reached in the subsequent binary logistic regression analysis (adjusted OR 1.27 (0.79 to 2.03), *P *= 0.327). These data are in some agreement with most of the recent studies demonstrating either a trend or a significant benefit for MTH in patients with VF/pVT and non-VF [43]. Very importantly, most of the published data did not undergo adjustment for multiple independent predictors, thus interpretation and comparison with our results is difficult. Our results may thus have a considerable heuristic value, and therefore additional international resuscitation registries should be encouraged to consider the same question with their data.

### Limitations

The GRR is based on voluntary participation of emergency services and hospitals. The registry cannot provide a complete picture of the total Germany-wide incidents of sudden cardiac arrest and resuscitation attempts at all. There is thus some degree of uncertainty with regard to representativeness of the register, but the GRR still reflects current practice throughout the country in both rural areas and big cities with different emergency medical system patterns. Nevertheless, voluntary registration and documentation by 23 medical emergency systems providing data for both the Preclinical care and Postresuscitation care datasets is probably associated with the risk of inclusion bias in the present study. But this problem is related to most of the published registries. For instance, the National Registry of Cardiopulmonary Resuscitation was started in 2000 as an international database of in-hospital resuscitation events worldwide, but it covers much less than 10% of potential hospitals. Further, the recent Parisian Region Out of Hospital Cardiac Arrest registry involved 68% patients with VF as initial rhythm, suggesting that there was also a highly selected cohort studied and a reasonable inclusion bias [18].

A total of 584 patients could be included in the present study, which may look like a rather small group; the reason for this number, however, was strict limitation to patients with complete Preclinical care and Postresuscitation care datasets, which resulted in a huge number of excluded patients. One of the main limitations of the present study is the selection bias for patients subjected to coronary intervention and hypothermia. Choice of postresuscitation therapeutic management was based on individual in-hospital postresuscitation treatment algorithms, so a bias in the selection of patients receiving any therapeutic option is highly likely. In addition, a substantial number of in-hospital variables that could influence survival and neurological outcome were not available in the database. These include body temperature management (for example, type of cooling induction, type of cooling device, surface vs. intravascular, target temperature), laboratory test levels, medications used, and details of revascularization procedures (for example, 'Thrombolysis In Myocardial Infarction' (TIMI) flow, type of stents, type of infarct, event-to-needle time). In addition, the present study does not differentiate between primary patient transports by the emergency medical system to the participating hospital or secondary transfer from one hospital to a hospital providing 24-hour coronary intervention services. Finally, our registry analysis is obviously limited by the nonrandomized and observational design, which contained no control group.

## Conclusions

The present study revealed potential beneficial effects on patient outcome for MTH and, in particular, primary PCI after successful resuscitation from OHCA. PCI was independently associated with good neurological outcome at hospital discharge. In addition, MTH was significantly associated with better neurological outcome at hospital discharge, although subsequent binary logistic regression analysis did not show statistical significance for MTH as an independent predictor in addition to PCI for good neurological outcome. Consequently, postresuscitation care on the basis of standardized protocols comprising PCI and MTH may be most beneficial and might therefore be considered for as many patients as possible. One of the main limitations of the present study may be the selection bias for patients subjected to coronary intervention and hypothermia. Finally, prospective randomized controlled studies are needed to elucidate potentials and limitations of a broader therapeutic use of PCI and hypothermia after successful CPR.

## Key messages

• Primary percutaneous coronary intervention was associated with good neurological outcome at hospital discharge after successful cardiopulmonary resuscitation as an independent factor.

• Mild therapeutic hypothermia was associated with increased chance of 24-hour survival as an independent factor.

• In terms of neurological outcome, mild therapeutic hypothermia tended to be associated with better neurological outcome although logistic regression analysis did not show statistical significance as an independent predictor.

• Postresuscitation care on the basis of standardized protocols including coronary intervention and mild therapeutic hypothermia may be beneficial after successful resuscitation.

• One of the main limitations of the present selected cohort registry study may be a selection bias for patients subjected to coronary intervention and hypothermia.

## Abbreviations

CPC: cerebral performance category; CPR: cardiopulmonary resuscitation; ECG: electrocardiogram; GRR: German Resuscitation Registry; MTH: mild therapeutic hypothermia; OHCA: out-of-hospital cardiac arrest; OR: odds ratio; PCI: percutaneous coronary intervention; pVT: pulseless ventricular tachycardia; ROSC: return of spontaneous circulation; VF: ventricular fibrillation.

## Competing interests

The authors declare that they have no competing interests.

## Authors' contributions

JTG and PM have made substantial contributions to conception and design, and drafted the manuscript. AC provided statistical support. BWB and TJ conceived of the study, and participated in its design and coordination and helped to draft the manuscript. JW, MM, TZ, BS, AB, and HF contributed data to the GRR and helped to revise the manuscript. JS and MF have been involved in the final revising the manuscript critically for important intellectual content, and have given final approval of the version to be published.

## Supplementary Material

Additional file 1 **Supplementary tables**. Table S1 presenting backwards stepwise binary logistic regression analysis for 24-hour survival in patients without coronary intervention (*n *= 430). Table S2 presenting backwards stepwise binary logistic regression analysis for good neurological outcome at hospital discharge in patients without coronary intervention (*n *= 430). Table S3 presenting backwards stepwise binary logistic regression analysis for 24-hour survival in normothermic patients (*n *= 405). Table S4 presenting backwards stepwise binary logistic regression analysis for good neurological outcome at hospital discharge in normothermic patients (*n *= 405). Table S5 presenting backwards stepwise binary logistic regression analysis for 24-hour survival in all patients (*n *= 584). Table S6 presenting backwards stepwise binary logistic regression analysis for good neurological outcome at hospital discharge in all patients (*n *= 584). Click here for file
