# Supplementary material for: Postresuscitation care with mild therapeutic hypothermia and coronary intervention after out-of-hospital cardiopulmonary resuscitation: a prospective registry analysis
Source: Crit Care. 2011 Feb 14;15(1):R61. doi: 10.1186/cc10035 (PMC3221994; doi:10.1186/cc10035)
Supplement: Additional file 1 — Supplementary tables. Table S1 presenting backwards stepwise binary logistic regression analysis for 24-hour survival in patients without coronary intervention (n = 430). Table S2 presenting backwards stepwise binary logistic regression analysis for good neurological outcome at hospital discharge in patients without coronary intervention (n = 430). Table S3 presenting backwards stepwise binary logistic regression analysis for 24-hour survival in normothermic patients (n = 405). Table S4 presenting backwards stepwise binary logistic regression analysis for good neurological outcome at hospital discharge in normothermic patients (n = 405). Table S5 presenting backwards stepwise binary logistic regression analysis for 24-hour survival in all patients (n = 584). Table S6 presenting backwards stepwise binary logistic regression analysis for good neurological outcome at hospital discharge in all patients (n = 584). [file cc10035-S1.DOC]

**Table S1: Backwards stepwise b**inary logistic regression analysis for 24h-survival in patients without coronary intervention (n=430)

|  |  | Regression coefficient | SE | OR | LCL | UCL | p value |
| --- | --- | --- | --- | --- | --- | --- | --- |
| Step 1 | Hypothermia | 2.128 | 0.341 | 8.395 | 4.306 | 16.367 | <0.001 |
|  | Fibrinolysis | -0.783 | 0.331 | 0.457 | 0.239 | 0.875 | 0.018 |
|  | Location at home | 0.360 | 0.225 | 1.433 | 0.923 | 2.225 | 0.109 |
|  | Age < 60 years | 0.322 | 0.256 | 1.379 | 0.835 | 2.277 | 0.209 |
|  | Witnessed OHCA | 0.035 | 0.247 | 1.035 | 0.638 | 1.681 | 0.889 |
|  | Bystander CPR | 1.245 | 0.338 | 3.473 | 1.792 | 6.730 | <0.001 |
|  | Cardiac etiology | -0.228 | 0.266 | 0.796 | 0.473 | 1.341 | 0.391 |
|  | VF/pVT as first rhythm | 0.765 | 0.255 | 2.150 | 1.304 | 3.544 | 0.003 |
|  | Constant | -0.382 | 0.329 | 0.683 |  |  | 0.246 |
| Step 2 | Hypothermia | 2.128 | 0.341 | 8.397 | 4.306 | 16.372 | <0.001 |
|  | Fibrinolysis | -0.778 | 0.330 | 0.459 | 0.241 | 0.876 | 0.018 |
|  | Location at home | 0.359 | 0.225 | 1.432 | 0.922 | 2.223 | 0.110 |
|  | Age < 60 years | 0.321 | 0.256 | 1.379 | 0.835 | 2.276 | 0.209 |
|  | Bystander CPR | 1.249 | 0.336 | 3.487 | 1.804 | 6.741 | <0.001 |
|  | Cardiac etiology | -0.228 | 0.266 | 0.796 | 0.472 | 1.341 | 0.391 |
|  | VF/pVT as first rhythm | 0.764 | 0.255 | 2.148 | 1.303 | 3.540 | 0.003 |
|  | Constant | -0.357 | 0.277 | 0.700 |  |  | 0.197 |
| Step 3 | Hypothermia | 2.136 | 0.341 | 8.467 | 4.344 | 16.505 | <0.001 |
|  | Fibrinolysis | -0.791 | 0.329 | 0.453 | 0.238 | 0.863 | 0.016 |
|  | Location at home | 0.351 | 0.224 | 1.421 | 0.916 | 2.204 | 0.117 |
|  | Age < 60 years | 0.372 | 0.249 | 1.450 | 0.890 | 2.362 | 0.135 |
|  | Bystander CPR | 1.235 | 0.336 | 3.439 | 1.781 | 6.642 | <0.001 |
|  | VF/pVT as first rhythm | 0.710 | 0.247 | 2.034 | 1.254 | 3.30 | 0.004 |
|  | Constant | -0.518 | 0.203 | 0.596 |  |  | 0.011 |
| Step 4 | Hypothermia | 2.109 | 0.339 | 8.239 | 4.237 | 16.021 | <0.001 |
|  | Fibrinolysis | -0.712 | 0.323 | 0.490 | 0.261 | 0.923 | 0.027 |
|  | Location at home | 0.352 | 0.224 | 1.422 | 0.917 | 2.203 | 0.116 |
|  | Bystander CPR | 1.295 | 0.334 | 3.650 | 1.898 | 7.020 | <0.001 |
|  | VF/pVT as first rhythm | 0.720 | 0.246 | 2.054 | 1.269 | 3.326 | 0.003 |
|  | Constant | -0.429 | 0.193 | 0.651 |  |  | 0.027 |
| Step 5 | Hypothermia | 2.109 | 0.339 | 8.240 | 4.243 | 16.003 | <0.001 |
|  | Fibrinolysis | -0.651 | 0.319 | 0.522 | 0.279 | 0.975 | 0.042 |
|  | Bystander CPR | 1.260 | 0.332 | 3.524 | 1.839 | 6.755 | <0.001 |
|  | VF/pVT as first rhythm | 0.675 | 0.243 | 1.964 | 1.219 | 3.163 | 0.006 |
|  | Constant | -0.229 | 0.144 | 0.112 | 0.795 |  |  |

OR indicates adjusted odds ratio; LCL, lower 95% confidence interval limit; UCL, upper 95% confidence interval limit; SE, standard error of the regression coefficient; VF, ventricular fibrillation; pVT, pulseless ventricular tachycardia; OHCA, out-of-hospital cardiac arrest; CPR, cardiopulmonary resuscitation. Backward elimination involved starting with all candidate variables (Step 1) and testing them one by one for statistical significance, deleting any that were not significant.

**Table S2: Backwards stepwise b**inary logistic regression analysis for good neurological outcome at hospital discharge in patients without coronary intervention (n=430)

|  |  | Regression coefficient | SE | OR | LCL | UCL | p value |
| --- | --- | --- | --- | --- | --- | --- | --- |
| Step 1 | Hypothermia | 0.804 | 0.314 | 2.235 | 1.209 | 4.132 | 0.01 |
|  | Fibrinolysis | -0.607 | 0.445 | 0.545 | 0.228 | 1.303 | 0.172 |
|  | Location at home | 0.542 | 0.317 | 1.719 | 0.924 | 3.197 | 0.087 |
|  | Age < 60 years | 0.771 | 0.326 | 2.161 | 1.14 | 4.097 | 0.018 |
|  | Witnessed OHCA | 0.287 | 0.358 | 1.332 | 0.661 | 2.686 | 0.423 |
|  | Bystander CPR | 0.341 | 0.401 | 1.406 | 0.641 | 3.083 | 0.395 |
|  | Cardiac etiology | -0.38 | 0.36 | 0.684 | 0.338 | 1.383 | 0.29 |
|  | VF/pVT as first rhythm | 0.971 | 0.329 | 2.641 | 1.386 | 5.033 | 0.003 |
|  | Constant | -2.875 | 0.465 | 0.056 |  |  | <0.001 |
| Step 2 | Hypothermia | 0.806 | 0.313 | 2.239 | 1.213 | 4.134 | 0.01 |
|  | Fibrinolysis | -0.57 | 0.443 | 0.565 | 0.237 | 1.346 | 0.197 |
|  | Location at home | 0.544 | 0.316 | 1.723 | 0.927 | 3.201 | 0.085 |
|  | Age < 60 years | 0.778 | 0.326 | 2.178 | 1.149 | 4.129 | 0.017 |
|  | Bystander CPR | 0.372 | 0.398 | 1.451 | 0.665 | 3.164 | 0.35 |
|  | Cardiac etiology | -0.352 | 0.358 | 0.703 | 0.349 | 1.417 | 0.325 |
|  | VF/pVT as first rhythm | 0.966 | 0.328 | 2.627 | 1.381 | 4.996 | 0.003 |
|  | Constant | -2.696 | 0.403 | 0.067 |  |  | <0.001 |
| Step 3 | Hypothermia | 0.789 | 0.312 | 2.201 | 1.195 | 4.054 | 0.011 |
|  | Fibrinolysis | -0.512 | 0.435 | 0.599 | 0.256 | 1.406 | 0.239 |
|  | Location at home | 0.522 | 0.315 | 1.685 | 0.909 | 3.120 | 0.097 |
|  | Age < 60 years | 0.83 | 0.321 | 2.294 | 1.224 | 4.299 | 0.01 |
|  | Cardiac etiology | -0.327 | 0.356 | 0.721 | 0.359 | 1.449 | 0.358 |
|  | VF/pVT as first rhythm | 0.99 | 0.327 | 2.692 | 1.420 | 5.106 | 0.002 |
|  | Constant | -2.668 | 0.402 | 0.069 |  |  | <0.001 |
| Step 4 | Hypothermia | 0.788 | 0.311 | 2.199 | 1.195 | 4.048 | 0.011 |
|  | Fibrinolysis | -0.528 | 0.433 | 0.59 | 0.252 | 1.380 | 0.223 |
|  | Location at home | 0.497 | 0.313 | 1.644 | 0.891 | 3.034 | 0.112 |
|  | Age < 60 years | 0.899 | 0.311 | 2.457 | 1.335 | 4.524 | 0.004 |
|  | VF/pVT as first rhythm | 0.898 | 0.308 | 2.454 | 1.342 | 4.487 | 0.004 |
|  | Constant | -2.875 | 0.34 | 0.056 |  |  | <0.001 |
| Step 5 | Hypothermia | 0.752 | 0.308 | 2.122 | 1.16 | 3.881 | 0.015 |
|  | Location at home | 0.462 | 0.31 | 1.587 | 0.864 | 2.916 | 0.137 |
|  | Age < 60 years | 0.821 | 0.304 | 2.274 | 1.253 | 4.125 | 0.007 |
|  | VF/pVT as first rhythm | 0.9 | 0.306 | 2.459 | 1.349 | 4.482 | 0.003 |
|  | Constant | -2.897 | 0.34 | 0.055 |  |  | <0.001 |
| Step 6 | Hypothermia | 0.758 | 0.307 | 2.134 | 1.168 | 3.898 | 0.014 |
|  | Age < 60 years | 0.81 | 0.303 | 2.249 | 1.242 | 4.072 | 0.007 |
|  | VF/pVT as first rhythm | 0.82 | 0.3 | 2.271 | 1.261 | 4.090 | 0.006 |
|  | Constant | -2.596 | 0.26 | 0.075 |  |  | <0.001 |

OR indicates adjusted odds ratio; LCL, lower 95% confidence interval limit; UCL, upper 95% confidence interval limit; SE, standard error of the regression coefficient; VF, ventricular fibrillation; pVT, pulseless ventricular tachycardia; OHCA, out-of-hospital cardiac arrest; CPR, cardiopulmonary resuscitation. Backward elimination involved starting with all candidate variables (Step 1) and testing them one by one for statistical significance, deleting any that were not significant.

**Table S3: Backwards stepwise binary logistic regression analysis for 24h-survival in normothermicpatients** (n=405)

|  |  | Regression coefficient | SE | OR | LCL | UCL | p value |
| --- | --- | --- | --- | --- | --- | --- | --- |
| Step 1 | PCI | 1.483 | 0.351 | 4.406 | 2.216 | 8.763 | <0.001 |
|  | Fibrinolysis | -1.233 | 0.354 | 0.292 | 0.146 | 0.584 | <0.001 |
|  | Location at home | 0.332 | 0.225 | 1.394 | 0.896 | 2.167 | 0.141 |
|  | Age < 60 years | 0.485 | 0.256 | 1.625 | 0.985 | 2.682 | 0.058 |
|  | Witnessed OHCA | 0.171 | 0.249 | 1.186 | 0.728 | 1.935 | 0.493 |
|  | Bystander CPR | 0.924 | 0.323 | 2.518 | 1.336 | 4.745 | 0.004 |
|  | Cardiac etiology | 0.062 | 0.276 | 1.064 | 0.620 | 1.826 | 0.821 |
|  | VF/pVT as first rhythm | 0.798 | 0.255 | 2.222 | 1.349 | 3.660 | 0.002 |
|  | Constant | -0.639 | 0.335 | 0.528 |  |  | 0.056 |
| Step 2 | PCI | 1.492 | 0.348 | 4.448 | 2.247 | 8.804 | <0.001 |
|  | Fibrinolysis | -1.229 | 0.354 | 0.293 | 0.146 | 0.586 | <0.001 |
|  | Location at home | 0.333 | 0.225 | 1.396 | 0.898 | 2.170 | 0.139 |
|  | Age < 60 years | 0.473 | 0.249 | 1.605 | 0.984 | 2.616 | 0.058 |
|  | Witnessed OHCA | 0.170 | 0.25 | 1.185 | 0.727 | 1.933 | 0.496 |
|  | Bystander CPR | 0.925 | 0.323 | 2.523 | 1.339 | 4.753 | 0.004 |
|  | VF/pVT as first rhythm | 0.812 | 0.248 | 2.252 | 1.386 | 3.659 | <0.001 |
|  | Constant | -0.593 | 0.268 | 0.552 |  |  | 0.027 |
| Step 3 | PCI | 1.505 | 0.347 | 4.506 | 2.281 | 8.901 | <0.001 |
|  | Fibrinolysis | -1.216 | 0.353 | 0.296 | 0.148 | 0.592 | <0.001 |
|  | Location at home | 0.331 | 0.225 | 1.392 | 0.896 | 2.164 | 0.141 |
|  | Age < 60 years | 0.475 | 0.249 | 1.608 | 0.987 | 2.621 | 0.057 |
|  | Bystander CPR | 0.945 | 0.322 | 2.573 | 1.370 | 4.835 | 0.003 |
|  | VF/pVT as first rhythm | 0.809 | 0.247 | 2.245 | 1.382 | 3.646 | <0.001 |
|  | Constant | -0.474 | 0.203 | 0.623 |  |  | 0.019 |
| Step 4 | PCI | 1.496 | 0.347 | 4.464 | 2.263 | 8.806 | <0.001 |
|  | Fibrinolysis | -1.155 | 0.35 | 0.315 | 0.159 | 0.625 | <0.001 |
|  | Age < 60 years | 0.462 | 0.248 | 1.587 | 0.976 | 2.582 | 0.063 |
|  | Bystander CPR | 0.917 | 0.32 | 2.501 | 1.335 | 4.688 | 0.004 |
|  | VF/pVT as first rhythm | 0.767 | 0.245 | 2.153 | 1.333 | 3.478 | 0.002 |
|  | Constant | -0.284 | 0.154 | 0.753 |  |  | 0.066 |

OR indicates adjusted odds ratio; LCL, lower 95% confidence interval limit; UCL, upper 95% confidence interval limit; SE, standard error of the regression coefficient; PCI, percutaneous coronary intervention; VF, ventricular fibrillation; pVT, pulseless ventricular tachycardia; OHCA, out-of-hospital cardiac arrest; CPR, cardiopulmonary resuscitation. Backward elimination involved starting with all candidate variables (Step 1) and testing them one by one for statistical significance, deleting any that were not significant.

**Table S4: Backwards stepwise binary logistic regression analysis for good neurological outcome at hospital discharge in normothermicpatients** (n=405)

|  |  | Regression coefficient | SE | OR | LCL | UCL | p value |
| --- | --- | --- | --- | --- | --- | --- | --- |
| Step 1 | PCI | 2.259 | 0.329 | 9.574 | 5.020 | 18.259 | <0.001 |
|  | Fibrinolysis | -1.037 | 0.504 | 0.355 | 0.132 | 0.953 | 0.040 |
|  | Location at home | 0.349 | 0.309 | 1.418 | 0.774 | 2.598 | 0.259 |
|  | Age < 60 years | 0.751 | 0.329 | 2.119 | 1.113 | 4.035 | 0.022 |
|  | Witnessed OHCA | 0.635 | 0.391 | 1.887 | 0.878 | 4.058 | 0.104 |
|  | Bystander CPR | -0.013 | 0.417 | 0.987 | 0.436 | 2.237 | 0.976 |
|  | Cardiac etiology | 0.316 | 0.415 | 1.372 | 0.608 | 3.094 | 0.446 |
|  | VF/pVT as first rhythm | 0.353 | 0.332 | 1.423 | 0.743 | 2.726 | 0.288 |
|  | Constant | -3.185 | 0.516 | 0.041 |  |  | <0.001 |
| Step 2 | PCI | 2.259 | 0.329 | 9.577 | 5.023 | 18.260 | <0.001 |
|  | Fibrinolysis | -1.037 | 0.504 | 0.354 | 0.132 | 0.951 | 0.039 |
|  | Location at home | 0.350 | 0.308 | 1.419 | 0.775 | 2.596 | 0.257 |
|  | Age < 60 years | 0.749 | 0.325 | 2.116 | 1.120 | 3.997 | 0.021 |
|  | Witnessed OHCA | 0.634 | 0.388 | 1.884 | 0.881 | 4.030 | 0.102 |
|  | Cardiac etiology | 0.317 | 0.415 | 1.372 | 0.609 | 3.094 | 0.445 |
|  | VF/pVT as first rhythm | 0.351 | 0.330 | 1.421 | 0.745 | 2.712 | 0.286 |
|  | Constant | -3.185 | 0.515 | 0.041 |  |  | <0.001 |
| Step 3 | PCI | 2.306 | 0.324 | 10.030 | 5.311 | 18.944 | <0.001 |
|  | Fibrinolysis | -1.035 | 0.505 | 0.355 | 0.132 | 0.955 | 0.040 |
|  | Location at home | 0.364 | 0.308 | 1.439 | 0.788 | 2.629 | 0.237 |
|  | Age < 60 years | 0.708 | 0.319 | 2.03 | 1.087 | 3.793 | 0.026 |
|  | Witnessed OHCA | 0.640 | 0.387 | 1.896 | 0.887 | 4.051 | 0.099 |
|  | VF/pVT as first rhythm | 0.413 | 0.320 | 1.512 | 0.807 | 2.832 | 0.197 |
|  | Constant | -2.969 | 0.424 | 0.051 |  |  | <0.001 |
| Step 4 | PCI | 2.280 | 0.322 | 9.779 | 5.207 | 18.364 | <0.001 |
|  | Fibrinolysis | -0.997 | 0.504 | 0.369 | 0.137 | 0.991 | 0.048 |
|  | Age < 60 years | 0.677 | 0.317 | 1.969 | 1.057 | 3.666 | 0.033 |
|  | Witnessed OHCA | 0.672 | 0.387 | 1.958 | 0.916 | 4.184 | 0.083 |
|  | VF/pVT as first rhythm | 0.382 | 0.318 | 1.465 | 0.785 | 2.732 | 0.230 |
|  | Constant | -2.770 | 0.385 | 0.063 |  |  | <0.001 |
| Step 5 | PCI | 2.380 | 0.312 | 10.806 | 5.860 | 19.928 | <0.001 |
|  | Fibrinolysis | -0.918 | 0.495 | 0.399 | 0.151 | 1.054 | 0.064 |
|  | Age < 60 years | 0.711 | 0.315 | 2.036 | 1.098 | 3.777 | 0.024 |
|  | Witnessed OHCA | 0.678 | 0.388 | 1.970 | 0.921 | 4.211 | 0.080 |
|  | Constant | -2.680 | 0.376 | 0.069 |  |  | <0.001 |

OR indicates adjusted odds ratio; LCL, lower 95% confidence interval limit; UCL, upper 95% confidence interval limit; SE, standard error of the regression coefficient; PCI, percutaneous coronary intervention; VF, ventricular fibrillation; pVT, pulseless ventricular tachycardia; OHCA, out-of-hospital cardiac arrest; CPR, cardiopulmonary resuscitation. Backward elimination involved starting with all candidate variables (Step 1) and testing them one by one for statistical significance, deleting any that were not significant.

**Table S5: Backwards stepwise b**inary logistic regression analysis for 24h-survival in all patients (n=584)

|  |  | Regression coefficient | SE | OR | LCL | UCL | p value |
| --- | --- | --- | --- | --- | --- | --- | --- |
| Step 1 | Hypothermia | 2.017 | 0.306 | 7.519 | 4.129 | 13.692 | <0.001 |
|  | PCI | 1.344 | 0.314 | 3.833 | 2.071 | 7.092 | <0.001 |
|  | Fibrinolysis | -1.006 | 0.304 | 0.366 | 0.202 | 0.663 | <0.001 |
|  | Location at home | 0.252 | 0.207 | 1.287 | 0.858 | 1.931 | 0.223 |
|  | Age < 60 years | 0.589 | 0.238 | 1.802 | 1.129 | 2.874 | 0.013 |
|  | Witnessed OHCA | 0.271 | 0.230 | 1.312 | 0.836 | 2.057 | 0.237 |
|  | Bystander CPR | 0.810 | 0.301 | 2.249 | 1.247 | 4.057 | 0.007 |
|  | Cardiac etiology | 0.000 | 0.254 | 0.999 | 0.608 | 1.643 | 0.997 |
|  | VF/pVT as first rhythm | 0.628 | 0.232 | 1.874 | 1.190 | 2.951 | 0.007 |
|  | Constant | -0.593 | 0.307 | 0.553 |  |  | 0.053 |
| Step 2 | Hypothermia | 2.017 | 0.306 | 7.519 | 4.131 | 13.686 | <0.001 |
|  | PCI | 1.343 | 0.312 | 3.832 | 2.081 | 7.059 | <0.001 |
|  | Fibrinolysis | -1.006 | 0.303 | 0.366 | 0.202 | 0.662 | <0.001 |
|  | Location at home | 0.252 | 0.207 | 1.287 | 0.858 | 1.931 | 0.223 |
|  | Age < 60 years | 0.589 | 0.233 | 1.802 | 1.141 | 2.846 | 0.012 |
|  | Witnessed OHCA | 0.271 | 0.230 | 1.312 | 0.836 | 2.057 | 0.237 |
|  | Bystander CPR | 0.810 | 0.301 | 2.249 | 1.247 | 4.055 | 0.007 |
|  | VF/pVT as first rhythm | 0.628 | 0.225 | 1.874 | 1.205 | 2.912 | 0.005 |
|  | Constant | -0.594 | 0.249 | 0.552 |  |  | 0.017 |
| Step 3 | Hypothermia | 2.016 | 0.306 | 7.504 | 4.123 | 13.661 | <0.001 |
|  | PCI | 1.363 | 0.310 | 3.909 | 2.127 | 7.183 | <0.001 |
|  | Fibrinolysis | -0.978 | 0.301 | 0.376 | 0.208 | 0.679 | <0.001 |
|  | Location at home | 0.252 | 0.207 | 1.286 | 0.858 | 1.928 | 0.223 |
|  | Age < 60 years | 0.590 | 0.233 | 1.804 | 1.143 | 2.846 | 0.011 |
|  | Bystander CPR | 0.841 | 0.299 | 2.320 | 1.290 | 4.172 | 0.005 |
|  | VF/pVT as first rhythm | 0.625 | 0.225 | 1.868 | 1.202 | 2.901 | 0.005 |
|  | Constant | -0.406 | 0.191 | 0.666 |  |  | 0.034 |
| Step 4 | Hypothermia | 2.015 | 0.305 | 7.501 | 4.124 | 13.645 | <0.001 |
|  | PCI | 1.357 | 0.310 | 3.883 | 2.114 | 7.133 | <0.001 |
|  | Fibrinolysis | -0.934 | 0.299 | 0.393 | 0.219 | 0.706 | 0.002 |
|  | Age < 60 years | 0.582 | 0.232 | 1.790 | 1.135 | 2.821 | 0.012 |
|  | Bystander CPR | 0.820 | 0.299 | 2.270 | 1.264 | 4.076 | 0.006 |
|  | VF/pVT as first rhythm | 0.594 | 0.223 | 1.811 | 1.171 | 2.803 | 0.008 |
|  | Constant | -0.261 | 0.149 | 0.770 |  |  | 0.079 |

OR indicates adjusted odds ratio; LCL, lower 95% confidence interval limit; UCL, upper 95% confidence interval limit; SE, standard error of the regression coefficient; PCI, percutaneous coronary intervention; VF, ventricular fibrillation; pVT, pulseless ventricular tachycardia; OHCA, out-of-hospital cardiac arrest; CPR, cardiopulmonary resuscitation. Backward elimination involved starting with all candidate variables (Step 1) and testing them one by one for statistical significance, deleting any that were not significant.

**Table S6: Backwards stepwise b**inary logistic regression analysis for good neurological outcome at hospital discharge in all patients (n=584)

|  |  | Regression coefficient | SE | OR | LCL | UCL | p value |
| --- | --- | --- | --- | --- | --- | --- | --- |
| Step 1 | Hypothermia | 0.235 | 0.241 | 1.265 | 0.789 | 2.030 | 0.329 |
|  | PCI | 1.641 | 0.248 | 5.160 | 3.171 | 8.395 | <0.001 |
|  | Fibrinolysis | -0.442 | 0.337 | 0.643 | 0.332 | 1.244 | 0.19 |
|  | Location at home | 0.033 | 0.233 | 1.033 | 0.655 | 1.631 | 0.888 |
|  | Age < 60 years | 1.212 | 0.244 | 3.361 | 2.082 | 5.424 | <0.001 |
|  | Witnessed OHCA | 0.645 | 0.303 | 1.906 | 1.053 | 3.451 | 0.033 |
|  | Bystander CPR | -0.338 | 0.313 | 0.713 | 0.386 | 1.317 | 0.280 |
|  | Cardiac etiology | 0.28 | 0.324 | 1.323 | 0.701 | 2.496 | 0.388 |
|  | VF/pVT as first rhythm | 0.42 | 0.246 | 1.522 | 0.940 | 2.467 | 0.088 |
|  | Constant | -3.016 | 0.411 | 0.049 |  |  | <0.001 |
| Step 2 | Hypothermia | 0.235 | 0.241 | 1.265 | 0.788 | 2.030 | 0.330 |
|  | PCI | 1.639 | 0.248 | 5.160 | 3.168 | 8.374 | <0.001 |
|  | Fibrinolysis | -0.437 | 0.335 | 0.646 | 0.335 | 1.246 | 0.192 |
|  | Age < 60 years | 1.210 | 0.244 | 3.352 | 2.080 | 5.402 | <0.001 |
|  | Witnessed OHCA | 0.648 | 0.302 | 1.911 | 1.057 | 3.456 | 0.032 |
|  | Bystander CPR | -0.343 | 0.311 | 0.710 | 0.385 | 1.307 | 0.271 |
|  | Cardiac etiology | 0.281 | 0.324 | 1.325 | 0.702 | 2.498 | 0.385 |
|  | VF/pVT as first rhythm | 0.417 | 0.245 | 1.517 | 0.939 | 2.453 | 0.089 |
|  | Constant | -2.999 | 0.392 | 0.050 |  |  | <0.001 |
| Step 3 | Hypothermia | 0.236 | 0.241 | 1.266 | 0.790 | 2.030 | 0.327 |
|  | PCI | 1.682 | 0.243 | 5.376 | 3.336 | 8.664 | <0.001 |
|  | Fibrinolysis | -0.43 | 0.335 | 0.650 | 0.337 | 1.254 | 0.199 |
|  | Age < 60 years | 1.178 | 0.240 | 3.247 | 2.028 | 5.196 | <0.001 |
|  | Witnessed OHCA | 0.654 | 0.302 | 1.923 | 1.065 | 3.473 | 0.030 |
|  | Bystander CPR | -0.332 | 0.310 | 0.718 | 0.391 | 1.318 | 0.285 |
|  | VF/pVT as first rhythm | 0.466 | 0.239 | 1.594 | 0.998 | 2.545 | 0.051 |
|  | Constant | -2.806 | 0.317 | 0.060 |  |  | <0.001 |
| Step 4 | PCI | 1.722 | 0.240 | 5.597 | 3.496 | 8.961 | <0.001 |
|  | Fibrinolysis | -0.42 | 0.336 | 0.657 | 0.340 | 1.270 | 0.211 |
|  | Age < 60 years | 1.165 | 0.239 | 3.205 | 2.006 | 5.120 | <0.001 |
|  | Witnessed OHCA | 0.66 | 0.301 | 1.934 | 1.073 | 3.487 | 0.028 |
|  | Bystander CPR | -0.33 | 0.310 | 0.719 | 0.392 | 1.320 | 0.287 |
|  | VF/pVT as first rhythm | 0.499 | 0.236 | 1.648 | 1.038 | 2.617 | 0.034 |
|  | Constant | -2.751 | 0.311 | 0.064 |  |  | <0.001 |
| Step 5 | PCI | 1.714 | 0.240 | 5.549 | 3.470 | 8.874 | <0.001 |
|  | Fibrinolysis | -0.459 | 0.335 | 0.632 | 0.328 | 1.219 | 0.171 |
|  | Age < 60 years | 1.121 | 0.235 | 3.068 | 1.935 | 4.863 | <0.001 |
|  | Witnessed OHCA | 0.615 | 0.297 | 1.850 | 1.034 | 3.308 | 0.038 |
|  | VF/pVT as first rhythm | 0.48 | 0.235 | 1.616 | 1.019 | 2.561 | 0.041 |
|  | Constant | -2.737 | 0.309 | 0.065 |  |  | <0.001 |
| Step 6 | PCI | 1.733 | 0.239 | 5.655 | 3.542 | 9.028 | <0.001 |
|  | Age < 60 years | 1.054 | 0.229 | 2.868 | 1.831 | 4.493 | <0.001 |
|  | Witnessed OHCA | 0.603 | 0.297 | 1.828 | 1.022 | 3.269 | 0.042 |
|  | VF/pVT as first rhythm | 0.474 | 0.234 | 1.606 | 1.014 | 2.542 | 0.043 |
|  | Constant | -2.772 | 0.309 | 0.063 |  |  | <0.001 |

OR indicates adjusted odds ratio; LCL, lower 95% confidence interval limit; UCL, upper 95% confidence interval limit; SE, standard error of the regression coefficient; PCI, percutaneous coronary intervention; VF, ventricular fibrillation; pVT, pulseless ventricular tachycardia; OHCA, out-of-hospital cardiac arrest; CPR, cardiopulmonary resuscitation. Backward elimination involved starting with all candidate variables (Step 1) and testing them one by one for statistical significance, deleting any that were not significant.
